# Activation of Nrf2 at Critical Windows of Development Alters Tissue-Specific Protein *S*-Glutathionylation in the Zebrafish (*Danio rerio*) Embryo

**DOI:** 10.3390/antiox13081006

**Published:** 2024-08-19

**Authors:** Emily S. Marques, Emily G. Severance, Paige Arsenault, Sarah M. Zahn, Alicia R. Timme-Laragy

**Affiliations:** Department of Environmental Health Sciences, School of Public Health and Health Sciences, University of Massachusetts Amherst, Amherst, MA 01003, USAemsev128@gmail.com (E.G.S.);

**Keywords:** sulforaphane (SFN), *tert*-butylhydroquinone (tBHQ), Nrf2, redox stress, glutathionylation, *Danio rerio*, liver, pancreas

## Abstract

Activation of Nrf2—the master regulator of antioxidative response—at different stages of embryonic development has been shown to result in changes in gene expression, but the tissue-specific and downstream effects of Nrf2 activation during development remain unclear. This work seeks to elucidate the tissue-specific Nrf2 cellular localization and the downstream changes in protein *S*-glutathionylation during critical windows of zebrafish (*Danio rerio*) development. Wild-type and mutant zebrafish embryos with a loss-of-function mutation in Nrf2a were treated with two canonical activators, sulforaphane (SFN; 40 µM) or tert-butylhydroquinone (tBHQ; 1 µM), for 6 h at either pharyngula, hatching, or the protruding-mouth stage. Nrf2a protein and *S*-glutathionylation were visualized in situ using immunohistochemistry. At the hatching stage, Nrf2a protein levels were decreased with SFN, but not tBHQ, exposure. Exposure to both activators, however, decreased downstream *S*-glutathionylation. Stage- and tissue-specific differences in Nrf2a protein and *S*-glutathionylation were identified in the pancreatic islet and liver. Protein *S*-glutathionylation in Nrf2a mutant fish was increased in the liver by both activators, but not the islets, indicating a tissue-specific and Nrf2a-dependent dysregulation. This work demonstrates that critical windows of exposure and Nrf2a activity may influence redox homeostasis and highlights the importance of considering tissue-specific outcomes and sensitivity in developmental redox biology.

## 1. Introduction

Glutathione (GSH), the primary cellular redox buffer in eukaryotes, is required for early-life vertebrate development and plays important roles in cell fate decisions. As reviewed by Hansen and Harris [1], a more reduced redox state generally promotes cell proliferation and survival, while a more oxidized state promotes cell differentiation and apoptosis. Perturbation of GSH homeostasis involving reactive oxygen species (ROS) during development has been linked to several mechanisms of teratogenesis in mouse, rat, and rabbit models [2]. Less severe perturbations to embryonic GSH can lead to subtle but persistent structural changes that can predispose individuals to diseases that emerge at later life stages, but in order to identify such agents, a fundamental understanding of stage- and tissue-specific developmental redox biology is needed. This study examines the role of a redox-responsive transcription factor that regulates many of the enzymes involved in GSH homeostasis, including nuclear factor erythroid 2-related factor 2 (NFE2L2; Nrf2) and a GSH-specific post-translational modification to proteins, *S*-glutathionylation, following acute exposures to two canonical Nrf2 activators to capture the effects of Nrf2 activation at three different stages of embryonic development in zebrafish (*Danio rerio*).

The Nrf2 pathway is an important mediator of GSH homeostasis and is responsive to electrophilic stress, ER stress, and autophagy disruptions, among others [3,4]. Nrf2 is constitutively expressed, and under basal conditions, Kelch-like ECH-associated protein 1 (Keap1), an adaptor subunit of the Cullin 3-based E3 ubiquitin ligase, binds Nrf2 and targets it for ubiquitination. Under oxidative conditions, cysteine residues on Keap1 are modified. This impedes the ubiquitination process and stabilizes Nrf2; thus, de novo Nrf2 can proceed unhindered to the nucleus, where Nrf2 dimerizes with small Maf proteins and binds to antioxidant response elements (ARE) [5]. AREs are located in the promoter regions of genes encoding for enzymes involved in GSH synthesis, reactive oxygen species (ROS) elimination, phase 2 detoxification enzymes, and many others that combat xenobiotic-induced redox stress. Two Nrf2 activators used in this study, *tert*-butylhydroquinone (tBHQ) and sulforaphane (SFN), directly interact with the cysteine 151 residue of Keap1 to stabilize Nrf2 [6,7]. SFN is an antioxidant found in cruciferous vegetables, such as broccoli and Brussels sprouts, and has been evaluated as a cancer therapeutic in several clinical trials [8,9,10,11]. tBHQ is a food preservative used to prevent oxidative deterioration of oil, fat, and meat products; in addition to the activation of Nrf2, pro-oxidant activity has also been described for tBHQ [12]. The gene expression profiles of Nrf2 activation by SFN and tBHQ are well-studied [13,14,15]; however, the spatiotemporal aspects of Nrf2 protein expression and the downstream effects of Nrf2 activation by SFN and tBHQ remain unclear.

Zebrafish have been used to model changes in the embryonic GSH redox environment because of their rapid development and ease of exposure. Zebrafish also have duplicate paralogous copies of Nrf2 (Nrf2a and Nrf2b) that have undergone sub-functionalization and are co-orthologs to the mammalian Nrf2; Nrf2a regulates ARE-mediated gene expression similar to that found in mammals, while Nrf2b is a negative regulator of genes controlling cell cycle progression, cell division, and apoptosis [16]. The GSH redox potential (GSH *E_h_*) and concentration of GSH changes in coordination with developmental stages and events [17]. During the pharyngula stage, which starts 24 h post-fertilization (hpf) in zebrafish, GSH *E_h_* is oxidized and has low concentrations of GSH. During the hatching stage at 48 hpf, the GSH Eh is oxidized while the GSH concentration is increased. After hatching and throughout the protruding-mouth stage, beginning at 72 hpf, the GSH concentration is stable but *E_h_* shifts from a more oxidized to a reduced state [1,17]. Because of these dynamic fluctuations during development, embryos are inherently susceptible to exogenous sources of redox modulation, and this has been linked to teratogenesis, as well as dysregulated glucose levels in developing β cells, insulin resistance, and disruptions of gene signaling involved with liver growth and development [2,18,19,20]. Previous studies in our laboratory have found that 24, 48, and 72 hpf are critical windows that are sensitive to redox modulation in the zebrafish, and we have shown that tBHQ and SFN exposure at these timepoints can alter pancreatic β-cell morphogenesis that persists through at least 96 hpf [21,22].

In addition to buffering cellular redox conditions, GSH can regulate protein structure and function through protein *S*-glutathionylation; glutathionylation is a reversible post-translational modification where GSH is attached to a protein [23,24]. *S*-Glutathionylation is used to alter or disrupt protein function during times of oxidative stress when proteins can potentially be damaged by free radicals and also to store and transport GSH. ROS can differentially oxidize cysteine residues in target proteins, and *S*-glutathionylation may mitigate or mediate such damage. In addition, this post-translational modification adds a net negative charge that can lead to distinct structural and functional changes to a target protein. Because this process is reversible, *S*-glutathionylation can act as a biological redox switch and is integral in many critical oxidative signaling events such as cell survival and cell death. Because of its sensitivity to GSH homeostasis, protein *S*-glutathionylation has been used as a biomarker for oxidative stress [25]. As reviewed by Xiong et al. [26], alterations in GSH and its associated pathways have been linked with a number of human diseases and aging; however, the cause–effect relationships regarding *S*-glutathionylation and the involvement of oxidative stress are still unclear.

In the study herein, SFN and tBHQ inductions of Nrf2a activation are evaluated during the critical windows of zebrafish development defined by GSH content and Eh (pharyngula, hatching, and protruding-mouth stage). We evaluate the role of Nrf2a activation using a wild-type and mutant zebrafish line (nrf2a^fh318/fh318^) that contains a point mutation in the DNA binding domain [27]. This mutation impedes the transcriptional activity of Nrf2a while maintaining the other elements of the pathway such as binding to Keap1 in the cytosol. Nrf2a protein and protein *S*-glutathionylation are visualized in the zebrafish in situ to test the hypothesis that changes in Nrf2a activation and *S*-glutathionylation will be dependent on specific critical developmental windows and Nrf2a transcriptional activity. In addition to global effects within the embryo overall, we also report tissue-specific changes in Nrf2a protein levels and *S*-glutathionylation on pancreatic islet and liver.

## 2. Materials and Methods

### 2.1. Chemicals and Reagents

Sulforaphane (SFN; Catalog #S4441) was purchased from Millipore-Sigma (Burlington, MA, USA). tert-Butylhydroquinone (tBHQ; Catalog #50-196-7735), dimethyl sulfoxide (DMSO), paraformaldehyde (PFA), phosphate-buffered saline (PBS), methanol, and Tween-20 were purchased from Fisher Scientific (Pittsburgh, PA, USA). Chicken Anti-Rabbit IgG AlexaFluor 594 (Catalog #A-21442), AlexaFluor 568 tagged Streptavidin (Catalog #S11226), and Biotinylated Glutathione Ethyl Ester (BioGee; Catalog #G36000) were purchased from Invitrogen (Carlsbad, CA, USA). Vectashield Antifade Mounting Medium with DAPI was purchased from Vector Laboratories (Burlingame, CA, USA). Primers, RNAlater, and the GeneJET RNA Purification Kit were purchased from Thermo Fisher Scientific (Waltham, MA, USA). 2-Mercaptoethanol (BME) was purchased from MP Biomedicals (Solon, OH, USA). iQ SYBR Green Supermix and iScript cDNA Synthesis Kits were purchased from Bio-Rad (Hercules, CA, USA).

### 2.2. Fish Husbandry

Zebrafish (*Danio rerio*) maintenance and procedures were conducted in accordance with the Guide for the Care and the Use of Laboratory Animals of the National Institutes of Health and with approval from the University of Massachusetts Amherst Institutional Animal Care and Use Committee (Animal Welfare Assurance Number A3551-01). Homozygous wild-type (nrf2a^+/+^) and nrf2a^fh318/fh318^ [27] mutant embryos (originally generated through the TILLING mutagenesis Project; R01HD076585) were crossed with Tg(insa:eGFP) [28] on an AB strain background to facilitate in vivo observation of β-cells. Breeding tanks (9.5 L) containing 20 female and 10 male adult fish were maintained on an automated Aquaneering aquatic habitat (San Diego, CA, USA). The fish were kept at standard conditions (28.5 °C with a 14 h light, 10 h dark lighting cycle), and fed GEMMA Micro 300 (Skretting, Westbrook, ME, USA) twice daily. Embryos were collected within 1 h post-breeding, washed, screened via light microscopy for fertilization, and staged according to Kimmel et al. [29]. At 24 hpf, embryos were manually dechorionated and reared in borosilicate glass scintillation vials containing 1 mL Danieau’s solution [17 mM NaCl, 2 mM KCl, 0.12 mM MgSO_4_, 1.8 mM Ca(NO_3_)_2_, 1.5 mM HEPES, pH 7.6] per embryo.

### 2.3. Chemical Exposures

Nrf2a wild-type (WT) and Nrf2a *m*/*m* zebrafish were treated with 40 µM SFN or 1 µM tBHQ in 0.01% DMSO in Danieau’s solution for 6 h during the following key critical developmental windows: pharyngula stage (24 to 30 hpf), hatching stage (48 to 54 hpf), and protruding-mouth stage (72 to 78 hpf). The control treatment was DMSO at a final concentration of 0.01% DMSO, which has been shown to have no effects when used in the zebrafish embryo developmental toxicity assay [30]. Concentrations and the 6 h exposure time for SFN and tBHQ were chosen based on previous studies demonstrating induction of the Nrf2a-mediated transcriptional response in zebrafish embryos [21,31,32]. Six hours post-exposure, the embryos were fixed in 4% PFA for Nrf2a immunohistochemistry (IHC); a subset was preserved in RNAlater for qPCR experiments. Embryos to be evaluated for protein *S*-glutathionylation were washed after exposure and maintained in Danieau’s solution. To allow for the downstream impacts of Nrf2a activation on the GSH content and glutathionylation activity, 24 h after the initial exposures, embryos were incubated with 100 µM BioGee (a biotinylated GSH molecule) for 2 h before fixation in 4% PFA.

### 2.4. Immunohistochemistry

IHC was performed following protocols previously described [33]. Briefly, embryos were fixed in 4% PFA in PBS overnight at 4 °C, rinsed in 0.1% Tween-20 in PBS (PBST), and stored in 100% methanol at −20 °C. The samples were rehydrated using standard methanol-PBST gradients and then heated at 70 °C for 20 min for antigen retrieval. The samples were then permeabilized using ice-cold acetone and incubated in a blocking solution of 5% Sheep’s serum in PBST for 2 h at room temperature. After blocking, the samples were incubated with the previously characterized rabbit-anti-Nrf2a antibody (1:1000 in blocking solution) overnight at 4 °C. This antibody was generated by 21st Century Biochemicals (Marlboro, MA, USA) against zebrafish Nrf2a with the peptide sequence NMPMQETLDMNAFMKPST and was a generous gift from Dr. Mark Hahn at the Woods Hole Oceanographic Institution. It has been previously validated to recognize zebrafish Nrf2a specifically [34]. After incubating with the primary antibody, the samples were washed and incubated with Alexa-tagged anti-rabbit secondary antibody (1:5000 in blocking solution) overnight at 4 °C. The samples were washed with PBST and stored in Vectashield at 4 °C until imaging. For protein *S*-glutathionylation IHC, the secondary antibody was AlexaFluor 594 tagged Streptavidin (1:5000 in blocking solution). A subset of embryos was incubated only with the secondary antibodies to control for non-specific binding.

### 2.5. RNA Isolation and qRT-PCR Analysis

Pools of 30–40 embryos at the pharyngula stage, or 15–20 later-stage embryos, were preserved in RNAlater at −80 °C. The embryos were thawed, transferred to lysis buffer, and sonicated with an Emerson Industrial Branson Sonifier^®^ (Danbury, CT, USA). RNA isolation was conducted using GeneJET RNA Purification. RNA quantity and quality were evaluated with a BioDrop μLITE spectrophotometer (Cambridge, United Kingdom). RNA (500 ng) of each sample was used with the iScript cDNA synthesis kit. cDNA was diluted 1:9 with nuclease-free water and stored at −80 °C. qRT-PCR reactions contained 10 μL of 2X iQ SYBR Green Supermix, 300 nM each of forward and reverse primers (1.2 μL total), 4.8 μL nuclease-free water, and 4 μL cDNA. The samples were run using a CFX Connect Real-Time PCR Detection System (Bio-Rad, in triplicate for each gene). Traces were assessed for quality control parameters including the melt curve peak and amplification curve. The primers were as follows: glutathione S-transferase Pi (NM_131734 gstp; 5′-CGACTTGAAAGCCACCTGTGTC-3′ and 5′-CTGTCGTTTTTGCCATATGCAGC-3′), β2-Microglobulin (NM_131163 b2m; 5′-CTGAAGAACGGACAGGTTATGT-3′ and 5′-ACGCTGCAGGTATATTCATCTC-3′), and β-actin (NM_131031 actb; 5′-CAACAGAGAGAAGATGACACAGATCA-3′ and 5′-GTCACACCATCACCAGAGTCCATCAC-3′). Gstp gene transcription fold-change was calculated using the ΔΔCT method [35]. b2m was used as a housekeeping gene; its transcription did not change across exposure groups. The actb gene was used as a secondary housekeeping gene to verify transcription patterns.

### 2.6. Microscopy and Image Analysis

Embryos were imaged using confocal microscopy at the Institute for Applied Life Sciences Nikon Center of Excellence at the University of Massachusetts Amherst. Body tissue and islet imaging was performed using a Nikon A1R-SIMe (Nikon TiE stand with A1 Resonant Scanning Confocal and N-SIM Structured Illumination Super-Resolution). Livers were imaged using a Nikon A1SP (Nikon TiE stand with A1 Spectral Detector Confocal) (Nikon Instruments Inc., Melville, NY, USA). Both microscopes were equipped with 405 nm, 488 nm, 561 nm, and 640 nm laser lines, and images were taken on the TRITC (Nrf2a or BioGee), FITC (insulin), and DAPI (nuclei) channels using the same laser intensity and gain for each image. Islet and body images were acquired with a 10× objective, and a 40× objective was used to acquire Z-stacks of the islet. A 40× objective was also used to acquire images of the liver and gut. Sequence scanning was used to eliminate cross-channel fluorescence overlap. Fiji open-source software release 2.9.0 [36] was used to measure Nrf2a and BioGee fluorescent intensity. The presented images were flipped horizontally to reflect biological orientation. All images were blinded to investigators using Fiji’s Blind Analysis Tool plug-in before analysis. The Fiji heatmap module was used to show the relative fluorescence intensity of Nrf2a and BioGee labeling. Background fluorescent intensity was also measured and subtracted from fluorescence measurements of the fish tissue. A threshold was set using the FITC channel to measure β-cells in the islet and to define the region that was then queried for the mean fluorescent intensity of the TRITC channel (Nrf2a or BioGee). Image coloration of the islets and livers was adjusted post-processing to differentiate the Nrf2a and BioGee images such that images presenting the Nrf2a-TRITC channel are presented in red and the BioGee images are presented in purple.

### 2.7. Colocalization

Colocalization was measured with the Coloc-2 plug-in for Fiji [36,37], using 40× confocal images of the liver, and a representative image of the pancreatic islet from each embryo taken from the middle of the Z-stack to obtain the largest islet area to evaluate differences in Nrf2a localization between the two tissue types. The liver or pancreatic islet (FITC channel) were selected as ROIs on their respective images. The TRITC (Nrf2a) and DAPI (nuclei) channels were evaluated using the Coloc-2 plug-in to generate Pearson’s R-value. The Pearson’s R coefficient was then converted to a normally distributed Z-score using Fisher’s Z-Transformation, and the Z-scores were assessed for statistical significance.

### 2.8. Statistics

GraphPad Prism v8.4.0.671 (La Jolla, CA, USA) was used to perform statistical tests for significance including Analysis of Variance (ANOVA) tests, followed by a Fisher’s Least Significant Differences (LSD) post hoc Test. Significance was signified by *p* < 0.05. All experiments were repeated for a minimum of 5 biological replicates per group.

## 3. Results

### 3.1. Spatiotemporal Changes in Nrf2a Protein

#### 3.1.1. Overall Trends

**Figure 1:**
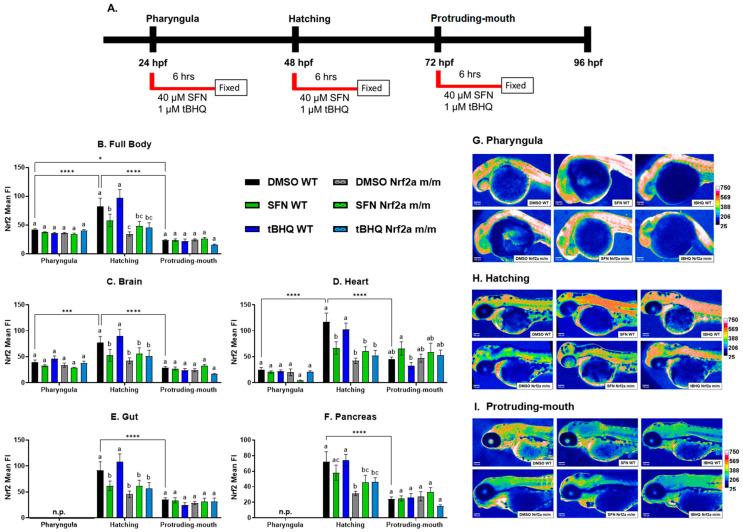
(**A**) Zebrafish were treated with 40 µM SFN or 1 µM tBHQ during the pharyngula, hatching, and protruding-mouth stages for 6 h and then fixed, and Nrf2a protein was labeled using immunohistochemistry (IHC). The mean fluorescence intensity (FI) of Nrf2a protein in the (**B**) body tissue, (**C**) brain, (**D**) heart, (**E**) gut, and (**F**) pancreas was measured; liver data are presented at higher magnification in Figure 3 below. Representative heatmaps were generated to visualize Nrf2a protein fluorescence differences at the (**G**) pharyngula, (**H**) hatching, and (**I**) protruding-mouth stages. The gut and pancreas are not present (n.p.) at the pharyngula stage. Statistics were performed with a two-way ANOVA and Fisher’s LSD post hoc test. *n* = 7–12 fish. Letters indicate significant differences (*p* ≤ 0.05) among groups. * *p* ≤ 0.05, *** *p* ≤ 0.001, **** *p* ≤ 0.0001. Scale bar = 100 µm.

Zebrafish were treated with 40 µM SFN or 1 µM tBHQ during the pharyngula, hatching, and protruding-mouth stage for 6 h and then fixed, and Nrf2a protein was labeled via IHC (Figure 1A). Nrf2a protein was increased in the body tissue (Figure 1B) at the hatching stage by 97% in the WT controls (DMSO) compared with the control WT embryos at the pharyngula stage. Nrf2a protein in the WT controls was decreased at the protruding-mouth stage by 44% and 72% compared with the pharyngula and hatching stages, respectively. Only during the hatching stage did Nrf2 activators and genotype impact Nrf2a protein in the body tissue. SFN, but not tBHQ exposure, decreased Nrf2a fluorescence by 29% in the WT embryos, and the control Nrf2a *m*/*m* embryos had lower Nrf2a fluorescence by 58% compared with the control WTs. Similar patterns were consistent in the brain (Figure 1C), heart (Figure 1D), gut (Figure 1E), and pancreas (Figure 1F). Overall, Nrf2a protein fluorescence was increased in the brain and heart in the control WT embryos at the hatching stage by 94% and 379%, respectively, compared with the pharyngula stage. Nrf2a protein fluorescence was decreased in the brain, heart, gut, and pancreas in the control WT embryos at the protruding-mouth stage by ~62–66% compared with the control WT embryos at the hatching stage. Exposure to SFN, but not tBHQ, during the hatching stage decreased Nrf2a fluorescence by 32%, 43%, and 33% in the brain, heart, and gut, respectively, compared with the control WT embryos. The control Nrf2a *m*/*m* embryos had decreased Nrf2a fluorescence in the brain, heart, gut, and pancreas by 45%, 64%, 50%, and 57%, respectively, compared with WT controls at the hatching stage. Representative heatmaps showing differences in Nrf2a protein expression generated from images of the embryos are shown in Figure 1G–I.

#### 3.1.2. Pancreatic Islet

**Figure 2:**
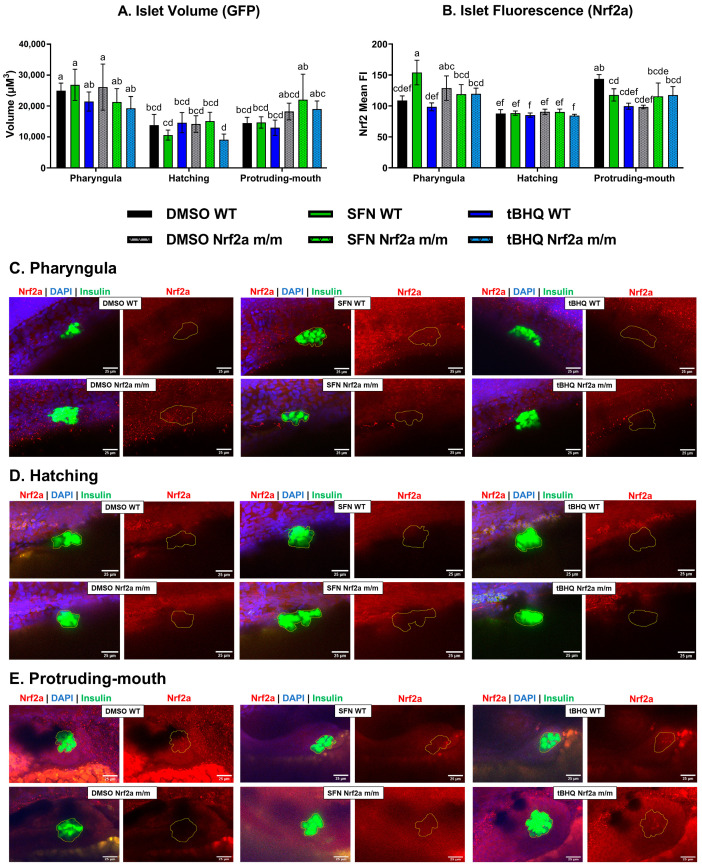
(**A**) Zebrafish were treated with 40 µM SFN or 1 µM tBHQ during the pharyngula, hatching, or protruding-mouth stage for 6 h and then fixed, and Nrf2a protein was labeled using immunohistochemistry (IHC). Z-stacks of the islet were acquired via confocal microscopy. (**A**) Islet volume and (**B**) islet mean fluorescence intensity (FI) of Nrf2a protein were measured with Nikon NIS elements software (available at Light Microscopy Core analysis workstations at the UMass Amherst Institute for Applied Life Sciences; https://www.microscope.healthcare.nikon.com/bioimaging-centers/nic-and-cofe/university-of-massachusetts-amherst). Representative images of each group at the (**C**) pharyngula, (**D**) hatching, and (**E**) protruding-mouth stage are shown. Images show max intensity projections of the islet Z-stack (circled in yellow) where FITC (green) represents β-cells, TRITC (red) represents Nrf2a protein, and DAPI (blue) represents nuclei. Statistics were performed with a two-way ANOVA and Fisher’s LSD post hoc test. *n* = 5–12 fish. Letters indicate significant differences (*p* ≤ 0.05) among groups. Scale bar = 25 µm.

To image the pancreatic islet, confocal Z-stacks under a 40× objective of the fixed fish were analyzed. The WT control embryos at the hatching and protruding-mouth stages had smaller islet volumes (Figure 2A) by 45% and 42%, respectively, compared with the WT controls at the pharyngula stage. There were no other significant differences in islet volume with treatment or genotype immediately after 6 h of treatment. The Nrf2a protein detection in the islet displayed stage-specific differences (Figure 2B), with the highest in the control WT embryos during the protruding-mouth stage by 32% and 64% compared with the WT controls at the pharyngula and hatching stages, respectively. Exposure during the pharyngula stage with SFN, but not tBHQ, increased Nrf2a detection in the islet by 42% in the WT embryos, but not in the Nrf2a *m*/*m* embryos. The islets of both genotypes were recalcitrant to changes in Nrf2a protein detection regardless of exposure or genotype at the hatching phase (Figure 2B). Exposure during the protruding-mouth stage with both SFN and tBHQ decreased Nrf2a fluorescence in the islet by 18% and 31% in the WT embryos, respectively. The control Nrf2a *m*/*m* embryos also had decreased detection by 32% compared with the control WT embryos at the protruding-mouth stage. Representative max intensity projections of the islet (GFP) display differences in Nrf2a protein fluorescence (red) generated from a 40× z-stack of the embryos at each timepoint (Figure 2C–E).

#### 3.1.3. Liver

**Figure 3:**
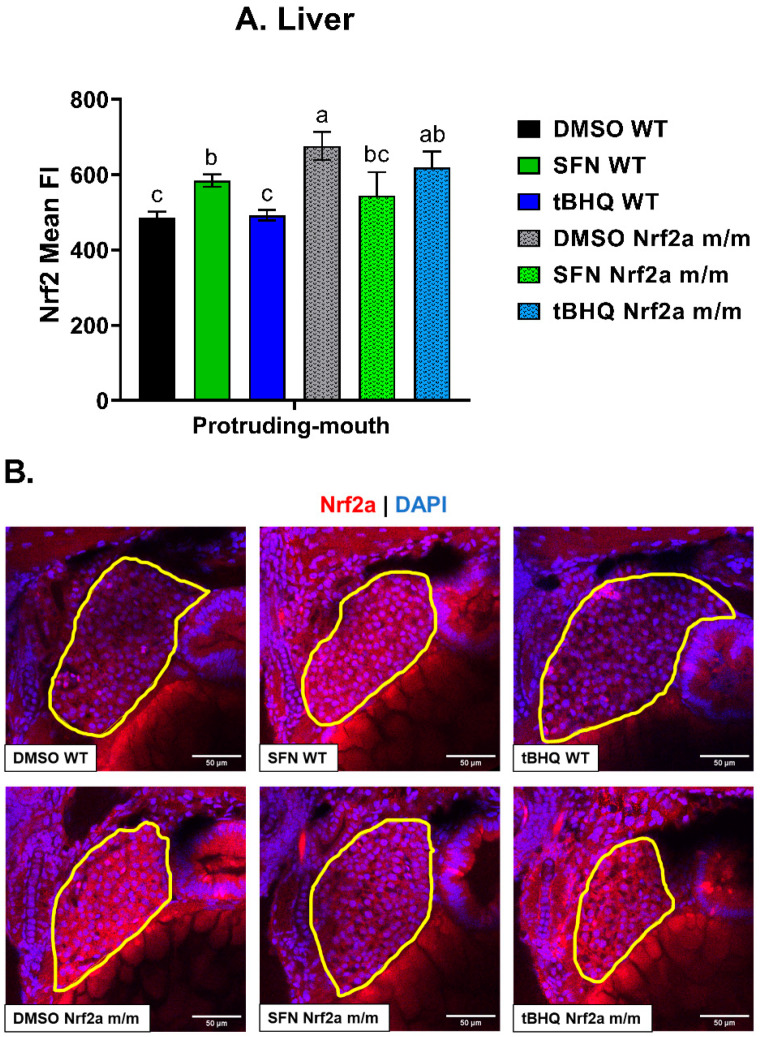
Zebrafish were treated with 40 µM SFN or 1 µM tBHQ during the protruding-mouth stage for 6 h and then fixed, and Nrf2a protein was labeled using immunohistochemistry (IHC). Liver images were acquired using confocal microscopy. (**A**) The mean fluorescence intensity (FI) of labeled Nrf2a protein in the liver was quantified via image analysis. (**B**) Representative images of the zebrafish liver are shown, where the liver is outlined in yellow, TRITC (red) represents Nrf2a, and DAPI (blue) represents nuclei. Statistics were performed using a one-way ANOVA followed by Fisher’s LSD post hoc test. *n* = 8–13 fish. Letters indicate significant differences (*p* ≤ 0.05). Scale bar = 50 µm.

To analyze Nrf2a protein content in the liver, confocal images of the fixed fish were taken under a 40× objective (Figure 3). Exposure to SFN, but not tBHQ, increased liver Nrf2a fluorescence by 20% in the WT embryos. The control Nrf2a *m*/*m* embryos had increased liver Nrf2a protein by 39% compared with the WT controls. Exposure to SFN in the Nrf2a *m*/*m* embryos decreased Nrf2a fluorescence in the liver by 20% compared with the control Nrf2a *m*/*m* embryos. Representative images of the livers (outlined in yellow) are shown in Figure 3B.

### 3.2. Colocalization

**Figure 4:**
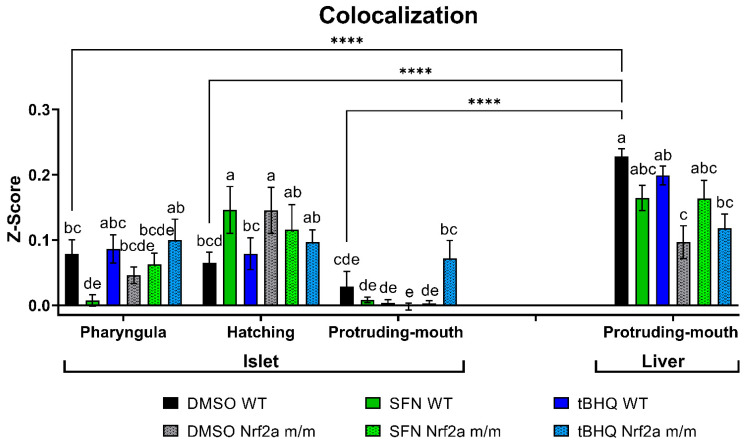
Zebrafish were treated with 40 µM SFN or 1 µM tBHQ during the pharyngula, hatching, and protruding-mouth stages for 6 h and then fixed, and Nrf2a protein was labeled using immunohistochemistry (IHC). Colocalization analysis was performed on 40× confocal images of the liver, and a representative image of the pancreatic islet was taken from the Z-stack. Pearson’s R coefficients were converted to normally distributed Z-scores, shown as means ± SEMs, where higher Z-scores indicate greater colocalization between Nrf2a protein and nuclear DAPI staining within the region of interest. Significance was assessed with a two-way or three-way ANOVA followed by Fisher’s LSD post hoc test. *n* = 5–13 fish. Different letters indicate significant differences (*p* ≤ 0.05) among tissues. **** *p* ≤ 0.0001 between islet and liver of the DMSO WT groups.

As Nrf2a accumulates in the nucleus when active, colocalization between anti-Nrf2a antibody labeling and DAPI nuclear staining was used to compare relative amounts of Nrf2a in the nucleus across the developmental stage, genotype, and treatment in the islet and liver (Figure 4). The WT control embryos did not have any significant changes in Nrf2a protein nuclear localization among the stages, but it did vary in the Nrf2a *m*/*m* control embryos, with the highest nuclear association found during the hatching stage. Nrf2a protein in the liver was more localized to the nucleus by almost 7-fold greater than in the islet at the protruding-mouth stage (Figure 4). In the islet at the pharyngula stage, nuclear Nrf2a protein localization was decreased by 91% with SFN exposure in the WT embryos. However, in the islet at the hatching stage, nuclear Nrf2a protein localization was increased by 125% with SFN exposure in the WT embryos, and nuclear Nrf2a protein localization was increased by 124% in the control Nrf2a *m*/*m* embryos compared with the control WT embryos. In the islet at the protruding-mouth stage, nuclear Nrf2a protein localization was slightly increased by 2-fold with tBHQ exposure in the Nrf2a *m*/*m* embryos compared with the control WT embryos (*p* = 0.1449) and significantly increased by ~10–40-fold with tBHQ treatment in the Nrf2a *m*/*m* embryos compared with the other treatment groups. In the liver at the protruding-mouth stage, nuclear Nrf2a protein localization was decreased by 28% with SFN exposure in the WT embryos, and nuclear Nrf2a protein localization was decreased by 58% in the control Nrf2a *m*/*m* embryos compared with the control WT embryos. Interestingly, liver nuclear Nrf2a protein localization was increased by 67% with SFN exposure in the Nrf2a *m*/*m* embryos compared with the control Nrf2a *m*/*m* embryos.

### 3.3. Protein S-Glutathionylation

#### 3.3.1. Overall Trends

**Figure 5:**
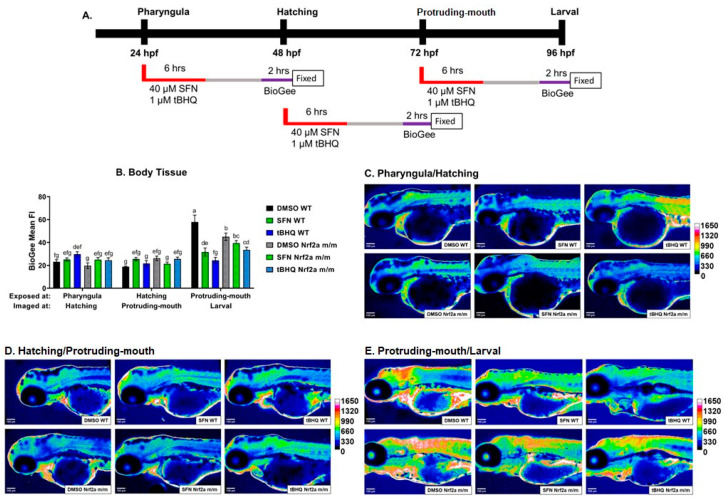
(**A**) Zebrafish were treated with 40 µM SFN or 1 µM tBHQ during the pharyngula, hatching, and protruding-mouth stages for 6 h and incubated with BioGee for 2 h prior to fixation (24 h post-exposure). BioGee protein conjugates were labeled in situ using immunohistochemistry. (**B**) Mean fluorescence intensity (Fl) of BioGee protein conjugates. Representative heatmaps were generated to visualize BioGee-protein conjugate fluorescence at the (**C**) pharyngula/hatching, (**D**) hatching/protruding-mouth, and (**E**) protruding-mouth/larval stages. Statistics were performed using a two-way ANOVA followed by Fisher’s LSD post hoc test. *n* = 8–10 fish. Different letters indicate significant differences (*p* ≤ 0.05) among groups. Scale bar = 100 μm.

To identify tissues with proteins undergoing *S*-glutathionylation downstream of Nrf2a activation, we incubated zebrafish embryos with a biotinylated GSH molecule (BioGee). Zebrafish were also treated with 40 µM SFN or 1 µM tBHQ during the pharyngula, hatching, and protruding-mouth stages for 6 h. Unlike the embryos used for Nrf2a expression changes, embryos were then washed and maintained in clean water, and 24 h after exposures began, the embryos were evaluated for downstream impacts on protein-*S*-glutathionylation. Embryos were incubated in 100 µM BioGee for 2 h and then immediately fixed, and protein *S*-glutathionylation was labeled via IHC (Figure 5A). The mean fluorescence intensity of protein *S*-glutathionylation was increased in the body tissue (Figure 5B) in embryos in the protruding-mouth/larval stage by 150% and 207% in the control WT group compared with the embryos in the pharyngula/hatching and hatching/protruding-mouth stages, respectively. Only with exposure to Nrf2 activators during the protruding-mouth stage did SFN and tBHQ in the WT embryos decrease protein *S*-glutathionylation at the larval stage by 45% and 58%, respectively. The control Nrf2a *m*/*m* embryos at the larval stage had lower protein *S*-glutathionylation by 22% compared with the control WT embryos. tBHQ exposure during the protruding-mouth stage in the Nrf2a *m*/*m* embryos also had decreased protein *S*-glutathionylation by 26% compared with the control Nrf2a *m*/*m* embryos at the larval stage. Representative heatmaps showing differences in BioGee labeling in the embryos are shown in Figure 5C–E.

#### 3.3.2. Pancreatic Islet

**Figure 6:**
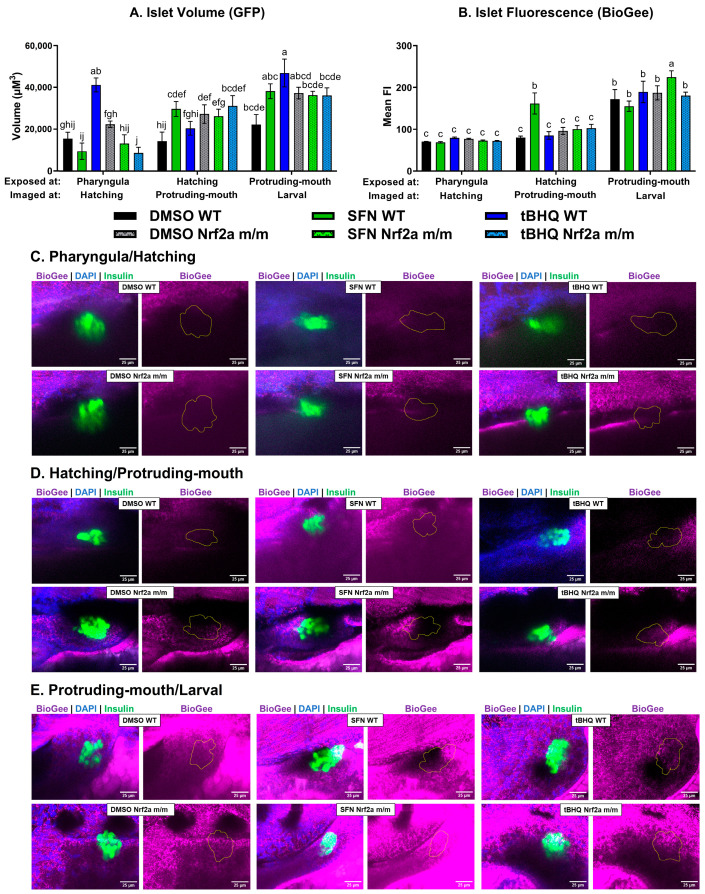
Zebrafish were treated with 40 µM SFN or 1 µM tBHQ for 6 h during the pharyngula, hatching, and protruding-mouth stages and incubated with BioGee for 2 h before fixation 24 h after the start of the exposure. BioGee protein conjugates were labeled in situ using IHC. Confocal microscopy was used to acquire Z-stacks of the islet. (**A**) Islet volume and (**B**) islet mean fluorescence intensity (FI) of BioGee. Representative images at the (**C**) pharyngula (start of exposure)/hatching (BioGee labeling and fixation), (**D**) hatching/protruding-mouth, and (**E**) protruding-mouth/larval stages are shown. Images are max intensity projections of the Z-stack the islet (outlined in yellow), where FITC (green) represents β-cells, TRITC (purple) represents BioGee protein conjugates, and DAPI (blue) represents nuclei. Statistical significance was assessed via two-way ANOVA followed by Fisher’s LSD post hoc test. *n* = 7–9 fish. Letters indicate significant differences (*p* ≤ 0.05) among groups. Scale bar = 25 µm.

To analyze protein S-glutathionylation in the pancreatic islet, confocal Z-stacks under a 40× objective of the treated fixed fish were analyzed. First, islet volume was impacted by both treatment, genotype, and exposure time with this exposure paradigm (Figure 6A). With exposures at the pharyngula stage, the tBHQ-exposed WT embryos had increased islet volume at the hatching stage by 167% compared with the WT control embryos, and the tBHQ-exposed Nrf2a *m*/*m* embryos had decreased islet volume at the hatching stage by 61% compared with the control Nrf2a *m*/*m* embryos. With exposures at the hatching stage, the SFN-exposed, but not tBHQ-exposed, WT embryos had increased islet volume at the protruding-mouth stage by 108% compared with the WT control embryos, and the Nrf2a *m*/*m* embryos had increased islet volume at the protruding-mouth stage by 91% compared with the control WT embryos. With exposures at the protruding-mouth stage, both the SFN- and tBHQ-exposed WT embryos had increased islet volume at the larval stage by 72% and 111%, respectively, compared with the WT control embryos, and the Nrf2a *m*/*m* embryos had increased islet volume at the larval stage by 68% compared with the WT control embryos. Overall, the WT control embryos at the larval stage had an increase in islet *S*-glutathionylation (Figure 6B) by 145% and 115%, compared to the WT control embryos at the hatching and protruding-mouth stages, respectively. Islet protein *S*-glutathionylation was increased by 102% at the protruding-mouth stage only with SFN exposure during the hatching stage in the WT embryos. Again, only SFN exposure during the protruding-mouth stage in the Nrf2a *m*/*m* embryos islet S-glutathionylation was increased by 26% and 16% at the protruding-mouth stage compared with the WT control and Nrf2a *m*/*m* embryos, respectively. Representative max intensity projections of the islet (GFP) display differences in BioGee fluorescence (purple) generated from 40× Z-stacks of the embryos at each timepoint (Figure 6C–E).

#### 3.3.3. Liver

**Figure 7:**
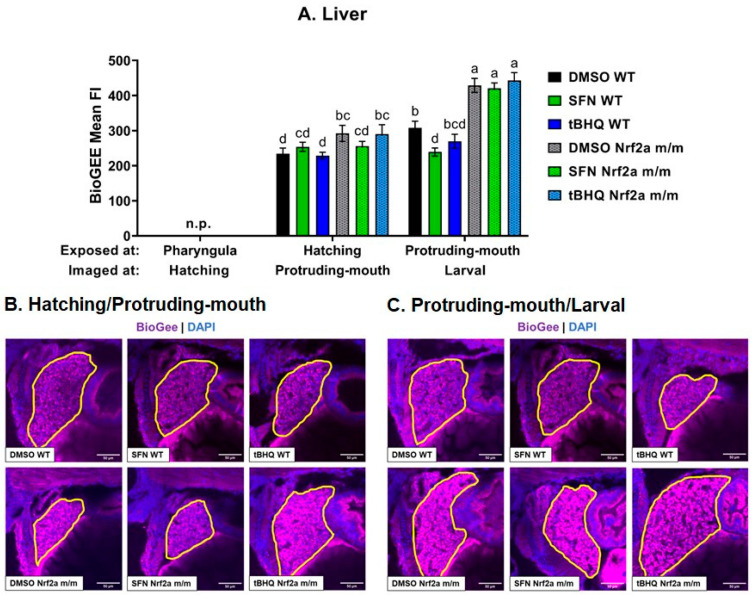
Zebrafish were treated with 40 µM SFN or 1 µM tBHQ during the hatching and protruding-mouth stages for 6 h and incubated with BioGee for 2 h before fixation 24 h after treatment. BioGee protein conjugates were labeled in situ using IHC. Confocal microscopy was used to acquire images of the liver using a 40× objective. (**A**) Liver mean fluorescence intensity (FI) of BioGee protein conjugates was determined via image analysis. Representative images of the zebrafish liver treated during the (**B**) hatching/protruding-mouth and (**C**) protruding-mouth/larval stage are shown where the liver is outlined in yellow, TRITC (purple) represents BioGee, and DAPI (blue) represents nuclei. The liver is not present (n.p.) at the stage visualized. Statistics were performed using a two-way ANOVA followed by Fisher’s LSD post hoc test. *n* = 6–12 fish. Different letters indicate significant differences (*p* ≤ 0.05) among groups. Scale bar = 50 µm.

To analyze protein *S*-glutathionylation in the liver, confocal images under a 40x objective of the treated fixed fish were analyzed (Figure 7). WT controls at the larval stage had an increase in liver *S*-glutathionylation by 31% compared with the WT controls at the protruding-mouth stage (Figure 7A). The Nrf2a *m*/*m* controls at the protruding-mouth stage had an increase in liver *S*-glutathionylation by 25% compared with the WT controls. Only SFN exposure during the protruding-mouth stage in the WT embryos decreased liver *S*-glutathionylation by 22% at the larval stage. Nrf2a *m*/*m* controls at the larval stage also had an increase in liver *S*-glutathionylation by 39% compared with the WT controls at the larval stage. Representative images of the livers are shown in Figure 3B.

### 3.4. qRT-PCR

**Figure 8:**
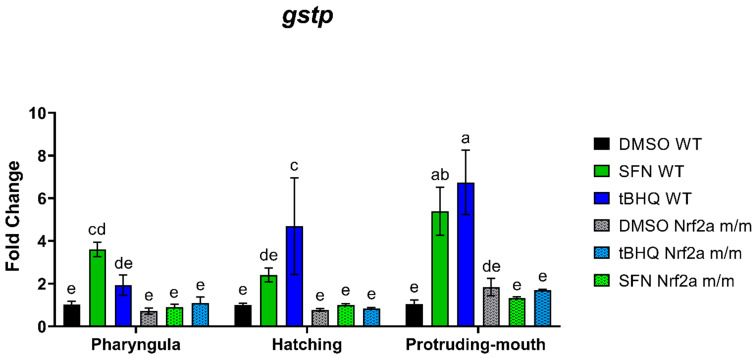
Zebrafish were treated with 40 µM SFN or 1 µM tBHQ for 6 h during the pharyngula, hatching, and protruding-mouth stages and then collected for gene expression via quantitative real-time PCR. mRNA expression was measured for *glutathione S-transferase Pi* (*gstp*) along with the housekeeping gene *β2-Microglobulin* (*b2m).* The ΔΔCT method was used to calculate fold change. Values are mean fold change ± SEM. Statistical significance was assessed via two-way ANOVA followed by Fisher’s LSD post hoc test. *n* = 2–3 pools of 15–20 (hatching and protruding-mouth stages) or 20–40 (pharyngula) fish. Different letters indicate significant differences (*p* ≤ 0.05) among groups.

To assess the transcriptional activity of Nrf2a, we used qRT-PCR to measure the expression of gstp as a bioindicator of exposure efficacy and Nrf2a activation expected in the WT but not the *m*/*m* embryos. The expression of gstp was increased with SFN and tBHQ exposure in the Nrf2a WT embryos but not the Nrf2a *m*/*m* embryos (Figure 8). SFN exposure in the WT embryos during the pharyngula stage increased gstp expression by 2.5-fold compared with the control embryos. Treatment with tBHQ in the WT embryos during the pharyngula stage only slightly increased gstp expression by 87% compared with the control embryos (*p* = 0.2742). tBHQ exposure in the WT embryos during the hatching stage increased gstp expression by 3.7-fold compared with the control embryos. SFN exposure in the WT embryos during the hatching stage only slightly increased gstp expression by 1.4-fold compared with the control embryos (*p* = 0.0904). Both the SFN- and tBHQ-exposed WT embryos during the protruding-mouth stage increased gstp expression by 4.2 and 5.5-fold, respectively. The expression of gstp was not altered by exposures in the Nrf2a *m*/*m* embryos.

## 4. Discussion

Embryos are highly susceptible to exogenous sources of redox modulation, and perturbation of redox balance has been linked to many adverse outcomes related to embryonic structures including the liver and pancreatic β-cells [2,18,19,20]. In this study, we used two canonical activators of Nrf2, i.e., SFN and tBHQ, during three critical windows of development to assess the impact on glutathionylation. To delineate the role of Nrf2a, the zebrafish co-orthologue of the mammalian Nrf2, we performed these exposures in a mutant zebrafish line (nrf2a^fh318/fh318^) containing a point mutation in the DNA binding domain [27] in comparison to WT embryos. Upon Nrf2 activation, Nrf2 protein accumulates and translocates to the nucleus where it can upregulate ARE-mediated genes such as *gstp*, a response that typically occurs within 6 h. Following transcriptional changes to glutathione-related and other Nrf2-mediated genes, there can be subsequent changes to the glutathione redox potential and in glutathionylation of proteins. To assess Nrf2a activation resulting from SFN and tBHQ exposure, embryos were examined immediately after exposure using Nrf2a protein IHC for nuclear localization and *gstp* expression. Protein *S*-glutathionylation was examined in fish 24 h after the initiation of exposure to SFN and tBHQ to link changes in Nrf2a activation at different developmental windows to downstream temporal alterations in glutathionylation. Here, we describe three windows of development in which Nrf2a protein and protein *S*-glutathionylation were modulated with Nrf2 activation and Nrf2a activity and tissue-specific differences between the pancreatic islet and liver, as summarized in Table 1. 

In Nrf2a *m*/*m* fish with little to no Nrf2a transcriptional activity, there was no change in *gstp* expression with either of the two Nrf2 activators, which is as expected as Nrf2 is one of the most important transcription factors recognized to stimulate the induction of many glutathione *S*-transferases genes (Gsts), including *gstp* [38]. This concurs with previous studies of SFN and tBHQ in Nrf2 knockout models in mice and zebrafish [39,40,41,42]. The use of Nrf2a *m*/*m* fish allows us to evaluate the specific role of Nrf2a signaling in the responses to Nrf2a activators and redox disruption while preserving the Nrf2a protein interactions with Keap1. It is important to note that the paralog Nrf2b also interacts with Keap1 and would be similarly activated in these exposure scenarios. However, as it has undergone subfunctionalization [16], genes responding to activated Nrf2b have been shown to be related to cell cycle maintenance and apoptosis. Additionally, Nrf2b functions to repress the transcription of these genes, making the response on glutathionylation and *gstp* expression specific to the action of Nrf2a.

Although GSH levels and the GSH redox state at various embryo stages have been evaluated previously [17], a limitation of this work is the actual levels of GSH and redox states were not measured in this study, and the data presented here with regards to glutathionylation are qualitative rather than quantitative. Still, these data do provide novel spatiotemporal information as to how embryos respond to Nrf2 activators at different developmental stages that are characterized by different glutathione parameters. At the pharyngula stage—with relatively low baseline levels of glutathione and an oxidized GSH Eh—there was no change in the amount of Nrf2a protein (Figure 1B), and no subsequent increase in total *S*-glutathionylation (Figure 5B) was observed in the Nrf2a mutants. In contrast, at the hatching stage—where baseline levels of glutathione are 2–3 times higher but have a similarly oxidized GSH Eh—there was a decrease in Nrf2a protein in the Nrf2a mutants (Figure 1B) but no change in subsequent *S*-glutathionylation (Figure 5B). At the protruding-mouth stage—with similar amounts of total glutathione but a more reduced GSH Eh than at the hatching stage—despite lower levels of basal Nrf2a protein than the prior stage, there was no change in Nrf2a protein in the Nrf2a mutants (Figure 1B). However, while there was a significant basal increase in total *S*-glutathionylation, the Nrf2a mutants had lower total *S*-glutathionylation (Figure 5B). This decrease in protein *S*-glutathionylation is unexpected in the mutants, as the mutants have little to no Nrf2a activity, so should be much more sensitive to oxidative stress [27]. This suggested that the decrease in protein *S*-glutathionylation in the mutants may be due to the control of protein *S*-glutathionylation by other compensatory regulators, or there may be an upregulation of deglutathionylation, catalyzed by glutaredoxin (Grx) [43], which warrants further study.

In the WT fish, Nrf2 activation by SFN and tBHQ during specific windows of development led to specific changes in *gstp* expression, Nrf2a protein expression, and protein *S*-glutathionylation in the overall body tissue. At the pharyngula stage, only SFN exposure led to a significant Nrf2a-mediated increase in gstp expression (Figure 8), but no change in the amount of Nrf2a protein (Figure 1B) and no subsequent increase in total *S*-glutathionylation (Figure 5B) were observed with either activator. In contrast, the hatching stage was the only stage where SFN exposure lowered total Nrf2a protein (Figure 1B), but there was still no change in subsequent *S*-glutathionylation with either SFN or tBHQ (Figure 5B). However, only tBHQ exposure led to a significant Nrf2a-mediated increase in *gstp* expression (Figure 8) at this stage. Exposure to both SFN and tBHQ at the protruding-mouth stage led to the largest increase in ARE-mediated gstp gene expression of the three stages (Figure 8). At this stage, despite lower levels of basal Nrf2a protein than in the prior two stages, there was no change in Nrf2a protein with either SFN or tBHQ (Figure 1B). Further, while there was a significant basal increase in total *S*-glutathionylation, exposure to both SFN and tBHQ at this stage led to a decrease in this measurement (Figure 5B). In comparison to our previous findings with another Nrf2 activator, dimethyl fumarate (DMF), SFN acted similarly to DMF, where DMF exposure at the hatching stage led to a decrease in Nrf2a protein followed by a subsequent decrease with *S*-glutathionylation [33]. This difference in Nrf2 expression by the different molecules may be due to differences in Nrf2 activation or activation by different regulators of Nrf2 expression, especially since pro-oxidant and anti-inflammatory activity has also been described for tBHQ [12,44]. As reviewed by Baird and Dinkova-Kostova [45], both tBHQ and SFN stabilize Nrf2 by binding to cysteine-151 on Keap1. However, there are known differences in Nrf2 regulation between the two inducers; tBHQ but not SFN is able to target Keap1 for ubiquitination [46]. Another potentially important difference is that SFN has been demonstrated to generate a mild increase in ROS and autophagosome and lysosome biogenesis via induction of nuclear translocation of transcription factor EB (TFEB) as an alternative cytoprotective mechanism of action [47]. SFN can also inhibit mTOR independent of Nrf2 by inhibiting HDAC6 and decreasing the catalytic activity of AKT [48], which may also be an explanation contributing to the different responses with these two Nrf2 activating chemicals. Another potential explanation is that changes in the endogenous developmental profile of gstp1 expression [15,17] may influence the *S*-glutathionylation response more so than the Nrf2-dependent upregulation of this gene. These findings collectively demonstrate the importance of the developmental baseline glutathione conditions in modifying the response to Nrf2 activation and underscore the complexity of interactions between Nrf2a and glutathione. As reviewed by Hansen et al. [1], the theory of redox regulation of development describes that the cellular process during normal development is coordinated by redox regulation and redox disruptions of normal redox signaling could produce adverse developmental outcomes; there is a clear need to understand the potential sensitivity to specific developmental pathways to support this theory. The goal of this work was to capture how Nrf2 activation during the key developmental windows could impact downstream redox regulation in the zebrafish embryo. A single, 6 h treatment was chosen for Nrf2 activation by SFN and tBHQ and to ensure sufficient time to induce Nrf2’s transcriptional response. Future work that incorporates later life stage timepoints would allow us to identify more persistent effects and if there are compensatory mechanisms to recover the redox state. In addition, different exposures that have persistent or transient, or greater or lesser, Nrf2 activation could provide more insight into these processes.

Further analysis of two redox-sensitive tissues—one with high expression of Nrf2 (the liver) and one with low endogenous expression of Nrf2 (pancreatic β-cells in the islet of Langerhans)—demonstrated the tissues-specific differences in the sensitivity to redox modulation. In the Nrf2a *m*/*m* fish, islet Nrf2a protein expression was increased at the pharyngula stage and decreased at the protruding-mouth stage (Figure 2B); however, there was no subsequent change in islet *S*-glutathionylation (Figure 6B). Unlike the islet, there was a compensatory increase in liver Nrf2a protein expression in the Nrf2a *m*/*m* fish at the protruding-mouth stage (Figure 3A) and the Nrf2a *m*/*m* fish had a subsequent increase in liver *S*-glutathionylation (Figure 7A). This indicates that the lack of Nrf2a activity in the liver induces oxidative stress conditions, and this may or may not be true of the islet.

Treatment with Nrf2 activators, i.e., SFN and tBHQ, also led to tissue-specific differences in the islet and liver. In the Nrf2a WT fish, islet Nrf2a protein expression was increased with SFN exposure at the pharyngula stage and decreased with both SFN and tBHQ exposures at the protruding-mouth stage (Figure 2B); however, only with SFN exposure at the hatching stage was there a subsequent increase in islet *S*-glutathionylation at the protruding-mouth stage (Figure 6B). Again, in contrast with the islet, liver Nrf2a protein expression was increased with SFN exposure at the protruding-mouth stage (Figure 3A), and exposure to both SFN and tBHQ at the protruding-mouth stage led to a subsequent decrease in liver *S*-glutathionylation at the larval stage (Figure 7A). This indicates that Nrf2 activation by both SFN and tBHQ, despite differences in Nrf2a protein levels, is protective from oxidative stress in the liver. It is well established that Nrf2 activation by SFN and tBHQ in hepatocytes and rodent models of liver injury is protective [49,50,51,52,53,54,55], and these results also suggest that even in the embryonic liver, Nrf2 may play a critical role in regulating liver injury and oxidative stress. The results in the islet were less clear and suggest a difference in tissue sensitivity to Nrf2 activation.

Previously, our lab showed that SFN and tBHQ negatively impacted islet morphology at 96 hpf, or the larval stage [21]. In this study, islet volume was measured immediately following treatment (when Nrf2a protein was measured; Figure 2A) and 24 h after exposure was initiated (when BioGee was measured; Figure 6A). Unsurprisingly, there was no difference between genotypes and exposure immediately following treatment (Figure 2A); however, 24 h after exposures were initiated, increased islet volume was observed with exposure to Nrf2 activators and embryos with the Nrf2a *m*/*m* genotype (Figure 6A). Exposure to tBHQ, but not SFN, at the pharyngula stage led to a subsequent increase in islet volume at the hatching stage. Exposure to SFN, but not tBHQ, at the hatching stage led to a subsequent increase in islet volume at the protruding-mouth stage. Lastly, exposure to both SFN and tBHQ at the protruding-mouth stage led to a subsequent increase in islet volume at the larval stage. The Nrf2a mutant fish at the protruding-mouth and larval stages also had an increase in islet volume. These results in the islet suggest that Nrf2 activation and the lack of Nrf2a activity are negatively impacting the islet. As demonstrated by Argaev-Frenkel and Rosenzweig [56], there is a delicate redox balance in insulin secretion and signaling, and mouse islets are sensitive to both oxidative and reductive stress. Further study of the specific opposing effects such as cell death and insulin secretion may help elucidate the specific role of Nrf2 in mediating these effects.

After observing these tissue-specific differences, colocalization of Nrf2a protein with DAPI staining was evaluated to identify nuclear localization (Figure 4). The observed differences with treatment and genotype with Nrf2a protein and DAPI colocalization corresponded to difference in Nrf2a protein expression. The islet Nrf2a nuclear colocalization was increased with SFN exposure and in the Nrf2a mutant fish at the hatching stage, which corresponds to observed decreases in Nrf2a protein expression in the body tissue (Figure 1B). The liver Nrf2a nuclear colocalization decreased with SFN exposure and in the Nrf2a mutant fish at the protruding-mouth stage, which corresponds to observed increases in Nrf2a protein expression in the liver (Figure 3A). These colocalization results only show the differences in Nrf2a localization but do not actually indicate Nrf2a activation. Further analysis using a tissue- or cell-specific measurement of Nrf2a activity is needed to confirm these results. Despite this, there was a clear difference between islet and liver cells (Figure 4), where the liver demonstrated more localization of Nrf2a protein to the nucleus, which is suggestive of higher basal Nrf2a activation and is supported by previous studies of Nrf2 activation in specific tissues [15]. Pancreatic islets, unlike the liver, which actively detoxifies xenobiotics, do not possess extensive antioxidant defense machinery. Additionally, ROS in β-cells play an important role in cellular signaling and insulin secretion [57,58]; thus, the liver and islet display some tissue-specific responses in terms of both sensitivity to Nrf2a activation and subsequent changes in glutathionylation.

## 5. Conclusions

This work demonstrated differences in Nrf2 activation and Nrf2a protein expression by the two chemical activators, i.e., SFN and tBHQ, in the zebrafish; however, both chemicals have similar effects on downstream protein *S*-glutathionylation. We identified the hatching stage in which GSH levels increase, where Nrf2a protein levels were modulated by Nrf2 activation and Nrf2a activity; however, it was only with exposures at the protruding-mouth stage and subsequent measurement at the larval stage where protein *S*-glutathionylation was modulated by Nrf2 activation and Nrf2a activity. This work also reported tissue-specific differences with Nrf2a expression and *S*-glutathionylation in the pancreatic islet and liver, which also highlights the importance of considering tissue-specific changes in developmental redox biology.

## Figures and Tables

**Table 1:** Summary of Nrf2a protein expression and protein *S*-glutathionylation at each exposure and timepoint.

**Exposure at the Pharyngula Stage**						
	**Nrf2a Mutants**			**Nrf2 Activators**		
	**Body Tissue**	**Liver**	**Islet**	**Body Tissue**	**Liver**	**Islet**
Nrf2a protein expression	No change	N/A	↑	No change	N/A	↑ SFN
*S*-glutathionylation	No change	N/A	No change	No change	N/A	No change
**Exposure at the Hatching Stage**						
	**Nrf2a Mutants**			**Nrf2 Activators**		
	**Body Tissue**	**Liver**	**Islet**	**Body Tissue**	**Liver**	**Islet**
Nrf2a protein expression	↑	N/A	No change	↓ SFN	N/A	No change
*S*-glutathionylation	No change	↑	No change	No change	No change	↑ SFN
**Exposure at the Protruding-Mouth Stage**						
	**Nrf2a Mutants**			**Nrf2 Activators**		
	**Body Tissue**	**Liver**	**Islet**	**Body Tissue**	**Liver**	**Islet**
Nrf2a protein expression	No change	↑	↓	No change	↑ SFN	↓ SFN ↓ tBHQ
*S*-glutathionylation	↑	↑	No change	↓ SFN ↓ tBHQ	↓ SFN	No change

↑—increased, ↓—decreased, N/A—tissue is not visable at the developmenial timepoint.

## Data Availability

Microscopy images and raw data are available upon request.
